# Towards the Enhancement in Photocatalytic Performance of Ag_3_PO_4_ Nanoparticles through Sulfate Doping and Anchoring on Electrospun Nanofibers

**DOI:** 10.3390/nano10050929

**Published:** 2020-05-11

**Authors:** Gopal Panthi, Kapil Raj Gyawali, Mira Park

**Affiliations:** 1 Department of Biomedical Sciences and Institute for Medical Science, Jeonbuk National University, Jeonju 54907, Korea; gopalpanthi2003@gmail.com; 2 Department of Chemistry, Birendra Multiple Campus, Tribhuvan University, Bharatpur 442000, Chitwan, Nepal; kapgyawali10@gmail.com

**Keywords:** electrospinning, SO_4_^2−^-Ag_3_PO_4_ nanoparticles, organic dyes, photocatalyst, visible light

## Abstract

Present work reports the enhancement in photocatalytic performance of Ag_3_PO_4_ nanoparticles through sulfate doping and anchoring on Polyacrylonitrile (PAN)-electrospun nanofibers (SO_4_^2−^-Ag_3_PO_4/_PAN-electrospun nanofibers) via electrospinning followed by ion-exchange reaction. Morphology, structure, chemical composition, and optical properties of the prepared sample were characterized using XRD, FESEM, FTIR, XPS, and DRS. The anchoring of SO_4_^2−^-Ag_3_PO_4_ nanoparticles on the surface of PAN-electrospun nanofibers was evidenced by the change in color of the PAN nanofibers mat from white to yellow after ion-exchange reaction. FESEM analysis revealed the existence of numerous SO_4_^2−^-Ag_3_PO_4_ nanoparticles on the surface of PAN nanofibers. Photocatalytic activity and stability of the prepared sample was tested for the degradation of Methylene blue (MB) and Rhodamine B (RhB) dyes under visible light irradiation for three continuous cycles. Experimental results showed enhanced photodegradation activity of SO_4_^2−^-Ag_3_PO_4_/PAN-electrospun nanofibers compared to that of sulfate undoped sample (Ag_3_PO_4_/PAN-electrospun nanofibers). Doping of SO_4_^2−^ into Ag_3_PO_4_ crystal lattice could increase the photogenerated electron–hole separation capability, and PAN nanofibers served as support for nanoparticles to prevent from agglomeration.

## 1. Introduction

The breakthrough work carried out by Yi et al. [1] opened a new door to engineer and synthesize silver phosphate (Ag_3_PO_4_)-based photocatalysts with enhanced performance that can find potential applications in dye photodegradation, hydrogen evolution, and killing microbes [2,3,4,5]. Ag_3_PO_4_ is a narrow band gap (2.36 eV) semiconductor material, which can generate reactive oxygen species (ROS) like OH^•^ or O_2_^• −^ from electron–hole pairs under visible light irradiation. Thus, generated ROS are responsible for its photocatalytic activities [6,7]. Application of Ag_3_PO_4_ as a visible light-driven photocatalyst is limited due to poor chemical stability, when applied in absence of sacrificial agent [1,8]. Therefore, successive investigations have been carried out for designing and fabricating Ag_3_PO_4_-based photocatalysts to overcome this limitation and improve their performance. In this regard, various studies have been reported, such as fabricating composites [9,10,11,12,13], coupling with other semiconductor materials [14,15] and doping suitable ions [16,17,18]. Out of these, doping of suitable ions into the crystal lattice of semiconductor materials could be an alternate strategy to enhance their photocatalytic property. It is reported that semiconductor material doped with suitable ions could prevent the recombination of photogenerated electron–hole pairs and consequently increases its stability and photocatalytic performance [19].

On that note, various results regarding the enhanced photocatalytic performance of Ag_3_PO_4_ via cations doping into its crystal lattice have been reported [20,21]. On the contrary, photocatalytic activities of Ag_3_PO_4_ doped with suitable anions have not been reported frequently. Meanwhile, a recent report has demonstrated the enhanced photocatalytic activity of sulfur-doped Ag_3_PO_4_ on the basis of hybrid density-functional calculation [22]; however, due to the strong P-O bond, doping of sulfur into Ag_3_PO_4_ crystal lattice seems more difficult. Therefore, instead of sulfur, SO_4_^2−^ might be a suitable anion as a dopant to replace PO_4_^3−^ from Ag_3_PO_4_ crystal lattice due to smaller radius of SO_4_^2−^ (0.218 nm) compared to that of PO_4_^3−^ (0.230 nm) [23,24]. On the other hand, use of photocatalyst nanoparticles in powder form creates a serious problem of agglomeration during photocatalysis, which leads to a reduction of surface area and ultimately, a decrease in photocatalytic performance [25]. In addition, the separation process of photocatalyst from solution becomes more difficult after use. To deal with these difficulties and avoid the loss of photocatalyst, polymer-electrospun nanofibers are being widely used as supports for nanoparticles [26,27,28]. Due to its excellent characteristics, like environmental stability, easy processability, and low density, PAN polymer is being extensively used for the fabrication of nanofibers as flexible support for photocatalyst nanoparticles using simple and versatile electrospinning technique [29,30].

Hence to realize the synergistic effect of sulfate-doped Ag_3_PO_4_ nanoparticles and PAN-electrospun nanofibers as support, our work is focused on the fabrication of SO_4_^2−^-Ag_3_PO_4_/PAN-electrospun nanofibers by combining electrospinning and ion-exchange reaction. Visible light photocatalytic activity of as fabricated sample was evaluated by observing photodegradation of MB and RhB dye solutions. Finally, we hope this visible-light-driven photocatalyst would be a promising candidate for the degradation of organic dyes from waste water to avoid negative effects to the dependent living ecology. To the best of our knowledge this type of work has not been reported so far.

## 2. Materials and Methods

### 2.1. Chemicals

The chemicals used in this work are Polyacrylonitrile (PAN, MW-150000, Sigma-Aldrich, St. Louis, MO, USA), Disodium hydrogen phosphate dihydrate (Na_2_HPO_4_.2H_2_O, Sigma-Aldrich, St. Louis, MO, USA), Silver nitrate (AgNO_3_, Sigma-Aldrich, St. Louis, MO, USA), Sodium sulfate (Na_2_SO_4_, Sigma-Aldrich, St. Louis, MO, USA), Methylene blue (MB, Sigma-Aldrich, St. Louis, MO, USA), Rhodamine B (RhB, Sigma-Aldrich, St. Louis, MO, USA) and *N, N*-dimethylformamide (DMF, SAMCHUN PURE CHEMICAL, Mogok-dong 117, Gyeonggi-d, Korea). All the chemicals were of AR grade and used without further purification. Distilled water was used to prepared aqueous solutions.

### 2.2. Fabrication of Na_2_HPO_4_/PAN Nanofiber

First, fine powder of Na_2_HPO_4_ (0.46 g) was dispersed in DMF (14 mL) and ultrasonicated for 1h. Then 1.5 g powder of PAN polymer was added to the above dispersion and magnetically stirred for 12h to prepare electrospinning solution. Electrospinning of the prepared solution was carried out by loading into a plastic syringe fitted with plastic micro-tip. The applied voltage and distance between needle tip to collector were set as 18 kV and 12 cm, respectively. The developing nanofibers were collected on rotating drum collector connected to DC motor. Thus obtained Na_2_HPO_4_/PAN-electrospun nanofibers were vacuum dried for 12 h at 70 °C.

### 2.3. Fabrication of SO_4_^2−^-Ag_3_PO_4_/PAN-Electrospun Nanofibers

**Scheme 1:**
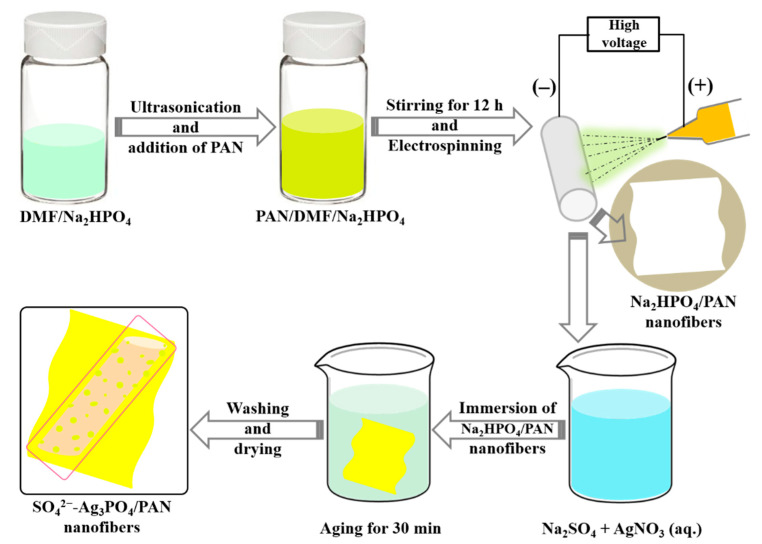
Schematic illustration for the fabrication of (SAP/PAN) SO_4_^2−^-Ag_3_PO_4_/PAN-electrospun nanofibers.

SO_4_^2−^-Ag_3_PO_4_/PAN-electrospun nanofibers were prepared following ion-exchange reaction. Briefly, Na_2_HPO_4_/PAN nanofiber mat (0.1 g) was immersed in 500 mL of AgNO_3_ solution containing SO_4_^2−^ ions for 30 min. Concentration of AgNO_3_ and Na_2_SO_4_ was maintained at 0.02 M and 0.01 M, respectively. Change in color of nanofibers mat from white to yellow during ion-exchange reaction indicated the anchoring of SO_4_^2−^-Ag_3_PO_4_ nanoparticles on the surface of PAN nanofibers. For comparison, Ag_3_PO_4_/PAN-electrospun nanofibers were also fabricated without adding SO_4_^2−^ ions into the AgNO_3_ solution under similar conditions. The resulting nanofibers mats were washed with deionized water and dried at 60 °C for 6 h before characterization. The schematic for the fabrication of SO_4_^2−^-Ag_3_PO_4_/PAN-electrospun nanofibers is illustrated in Scheme 1. For the convenience of description, different samples are named as pristine PAN, AP/PAN, and SAP/PAN corresponding to pristine PAN-electrospun nanofibers, Ag_3_PO_4_/PAN-electrospun nanofibers, and SO_4_^2−^-Ag_3_PO_4_/PAN-electrospun nanofibers, respectively.

### 2.4. Characterization

Crystalline nature of the samples was investigated using X-ray diffractometer (XRD, Empyrean, PANAlytical, Eindhoven 5651 GH, Netherlands) with Cu Kα (λ = 1.540 Å) radiation over Bragg angles ranging from 10° to 80°. Field emission scanning electron microscope (FESEM, GeminiSEM 500, Carl Zeiss Microscopy GmbH, 73447, Oberkochen, Germany) equipped with energy dispersive X-ray spectroscopy (EDS) was used to study the morphology and elemental composition of the samples. Bonding configuration of the samples was characterized applying Fourier-transform infrared (FT-IR, FT/IR-4200, Jasco international Co., Ltd., 4-21, Sennin-cho 2-chome, Hachioji, Tokyo 193-0835, Japan) through attenuated total reflectance mode (ATR). Furthermore, the surface element composition analysis of SO_4_^2−^-doped/undoped samples was studied using X-ray photoelectron spectroscopy (XPS, AXIS-NOVA, Kratos Analytical Ltd., Manchester, M17 1GP, UK), and the light absorption properties of the prepared samples was evaluated from UV-vis diffusive reflectance spectra (DRS) obtained from UV-vis spectrophotometer (UV-2600 240 EN, SHIMADZU CORPORATION, Kyoto, Japan).

### 2.5. Investigation of Photocatalytic Activity

To investigate the photocatalytic performance, as fabricated samples (pristine PAN, AP/PAN, and SAP/PAN) were utilized as visible-light-driven photocatalysts for the degradation of MB and RhB solutions at 10 ppm using a solar simulator having an internal xenon lamp (DYX300P, DYE TECH Co., Seoul, Korea) equipped with a UV cutoff filter. The experiments were carried out in a glass vial containing dye solution (50 mL) and photocatalyst (100 mg). Prior to irradiation, the suspension was magnetically stirred under dark conditions for 30 min to establish adsorption/desorption equilibrium. Afterward, visible light obtained from the 200-W xenon lamp was irradiated under continuous magnetic stirring. Aliquots were taken at regular time intervals (10 min) and the concentration of the dye solution was measured spectrophotometrically by recording the absorbance using a UV-vis spectrophotometer (HP 8453 UV–vis spectroscopy system, Hudson, MA, USA). The total organic carbon (TOC) content in residual solution was determined with a TOC analyzer (multi N/C 3100, Analytik Jena, Konrad-Zuse-Strasses 1 07745 Jena, Germany).

## 3. Results and Discussion

**Figure 1:**
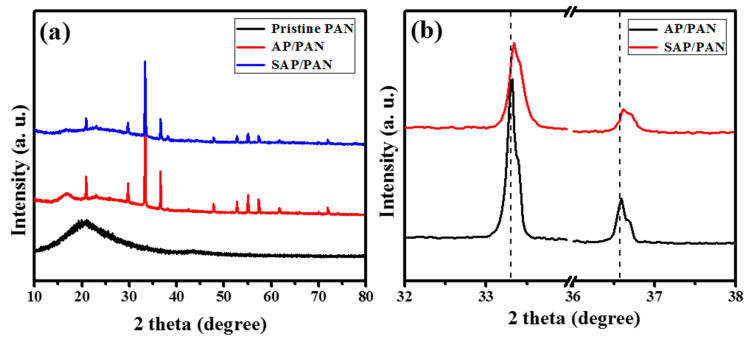
(**a**) XRD diffraction patterns—pristine PAN, AP/PAN, and SAP/PAN; and (**b**) magnified peaks of (210) and (211) crystal planes.

XRD analysis was applied to investigate the crystallinity and effect of sulfate doping into Ag_3_PO_4_ crystal lattice. Figure 1a displays XRD patterns of pristine PAN, AP/PAN, and SAP/PAN. A broad and noncrystalline peak at 2θ of 20–30° in all formulations was assigned to the (110) crystal plane of PAN polymer [29]. Besides, the diffraction peaks at 2θ of 20.89°, 29.69°, 33.31°, 36.51°, 42.42°, 47.74°, 52.66°, 55.10°, 57.29°, 61.63°, 65.71°, 70.06°, 71.89°, and 73.78° in AP/PAN and SAP/PAN were attributed to the crystal planes of (110), (200), (210), (211), (220), (310), (222), (320), (321), (400), (411), (420), (421), and (332) of Ag_3_PO_4_, respectively (JCPDS card No: 06-0505). Effect of sulfate doping into Ag_3_PO_4_ crystal lattice was examined by observing the magnified XRD patterns of AP/PAN and SAP/PAN (Figure 1b). The gradual shift of peaks corresponding to (210) and (211) crystal planes of SAP/PAN towards higher 2θ angle was observed. Such shifting might be attributed to the decrease in crystal lattice constant due to SO_4_^2−^ ions entering into Ag_3_PO_4_ crystal lattice by replacing PO_4_^3−^ ions since SO_4_^2−^ has a smaller ionic radius than that of PO_4_^3−^ [24,31].

**Figure 2:**
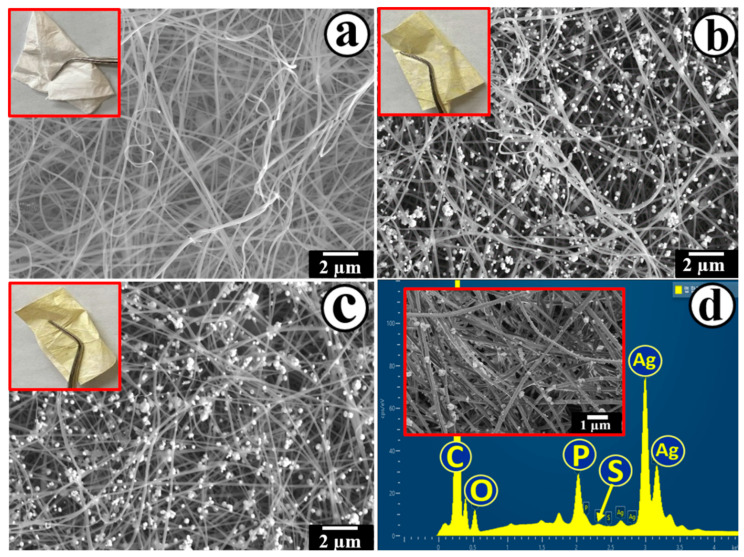
FESEM images; (**a**) Na_2_HPO_4_/PAN nanofibers, (**b**) AP/PAN, (**c**) SAP/PAN. Panel (**d**) represents the corresponding EDS of (**c**). Insets in Figure (**a**–**c**) are digital photograph of corresponding nanofibers and inset in Figure (**d**) is FESEM image of used SAP/PAN.

**Figure 3:**
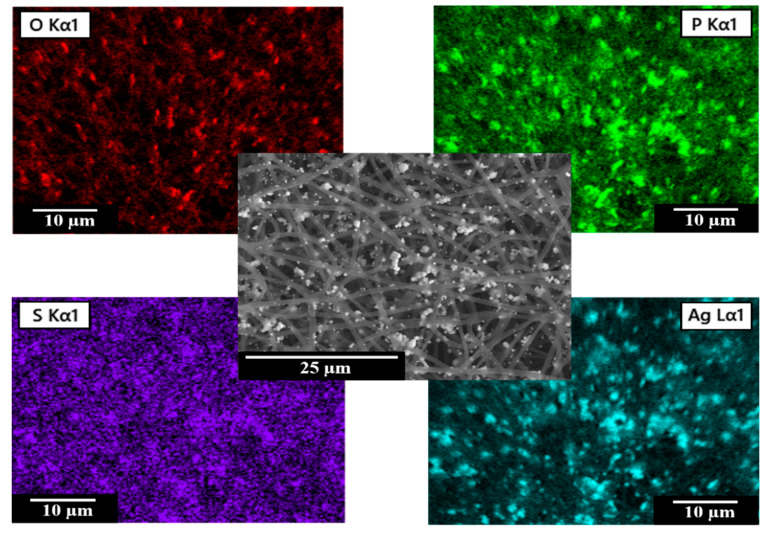
Elemental mapping of SAP/PAN.

Figure 2a–c show the typical FESEM images of Na_2_HPO_4_/PAN electrospun nanofibers, AP/PAN, and SAP/PAN fabricated by electrospinning. All samples exhibited bead-free, continuous, and randomly oriented nanofibers having an average diameter of 430 nm. Na_2_HPO_4_/PAN nanofibers could serve as both support and source to subsequent ion-exchange reaction to fabricate sulfate undoped/doped Ag_3_PO_4_/PAN nanofibers. After the growth of nanoparticles by ion-exchange reaction, the surface of nanofibers (AP/PAN) (Figure 2b) and (SAP/PAN) (Figure 2c) was no longer smooth compared to Na_2_HPO_4_/PAN nanofibers. The nanoparticles with some agglomerations were uniformly anchored on PAN nanofiber surface, which was evidenced through the color change of Na_2_HPO_4_/PAN nanofibers mate from white to yellow after ion-exchange reaction (insets Figure 2a−c). In order to elucidate the stability of photocatalyst, FESEM characterization of used SAP/PAN was performed. As depicted in the Figure (inset of Figure 2d), the used sample could keep its integrity even after three cycle tests without distinct loss of nanoparticles. Furthermore, elemental composition of SAP/PAN was investigated by FESEM-EDS (Figure 2d). The EDS spectra indicated the presence of considerable amount of C, O, P, S, and Ag in SAP/PAN without other impurities, justifying the sample being composed of sulfate-doped Ag_3_PO_4_ and PAN. Similarly, existence of sulfate-doped Ag_3_PO_4_ nanoparticles on PAN nanofibers was further confirmed, observing spatial distribution of O, P, S, and Ag elements by elemental mapping of SAP/PAN (Figure 3). As shown in the mapping images, all the elements are almost homogeneously distributed on PAN nanofiber surface, specifying the presence of sulfate-doped Ag_3_PO_4_ nanoparticles.

**Figure 4:**
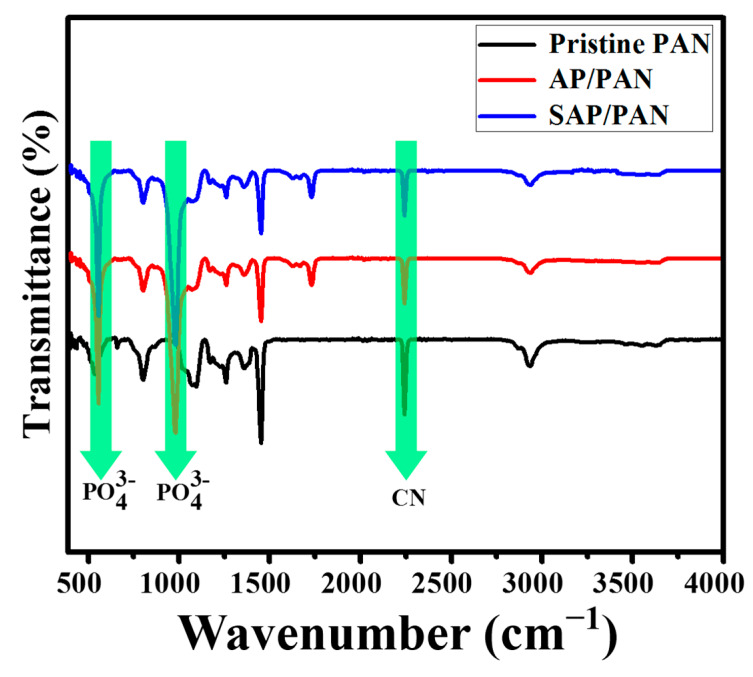
FTIR spectra; pristine PAN, AP/PAN, and SAP/PAN.

Figure 4 represents the FTIR spectra of pristine PAN, AP/PAN, and SAP/PAN. The absorption band centered at about 2243 cm^−1^ in all samples is assigned to the nitrile group (C≡N) of PAN. Similarly, all samples possessing characteristics bands attributed to aliphatic CH group vibrations of different modes in the methylene group of PAN were located in the regions 1220–1270 cm^−1^, 1350–1380 cm^−1^, 1450–1460 cm^−1^, and 2870–2931 cm^−1^ [32]. Moreover, the absorption bands located at about 1600 cm^−1^ and 3400–3500 cm^−1^ were assigned to stretching vibration of H-O-H and bending O-H to denote the presence of physically absorbed water molecules [33]. Furthermore, absorption bands located at about 550 cm^−1^ and 981 cm^−1^ in AP/PAN and SAP/PAN were due to the molecular vibration of PO_4_^3−^ [30,34]. However, the absorption band that locates at about 983 cm^−1^ [35,36] corresponding to SO_4_^2−^ in SAP/PAN was not apparent, which could be due to overlapping with the absorption band of PO_4_^3−^ or small amount of SO_4_^2−^. Hence, all these FTIR results suggested SO_4_^2−^-doped/undoped Ag_3_PO_4_ nanoparticles were immobilized on PAN nanofibers.

**Figure 5:**
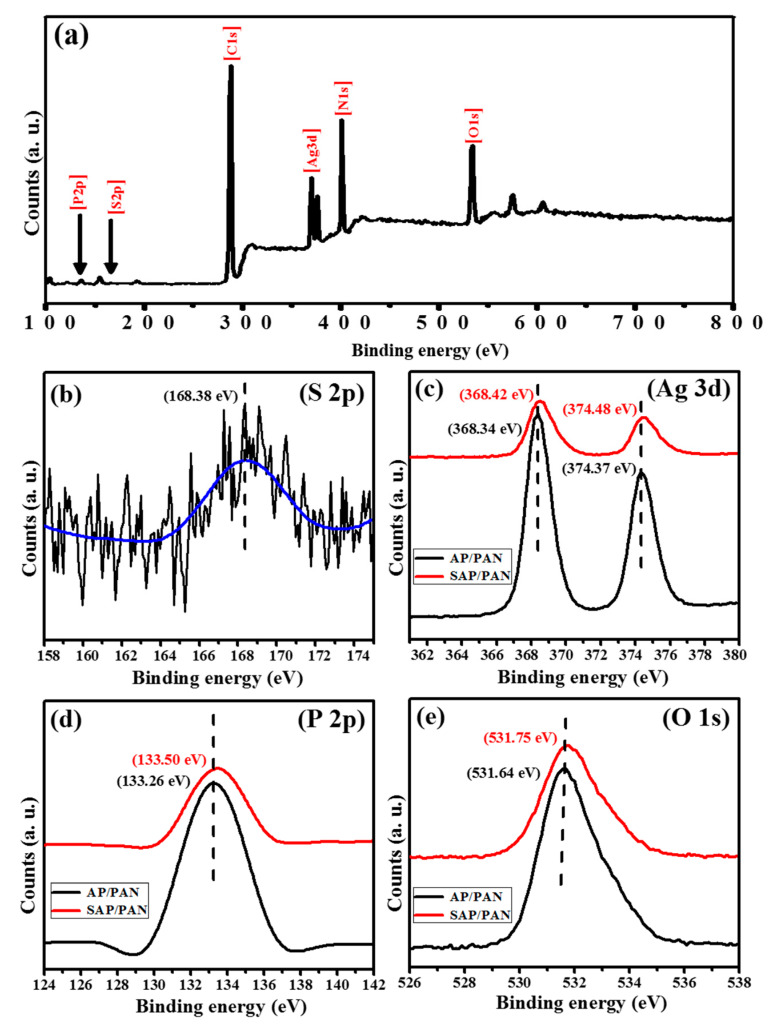
XPS spectra; survey spectrum of SAP/PAN (**a**). Panels (**b**–**e**) represent the high-resolution spectra of AP/PAN and SAP/PAN.

The coexistence of SO_4_^2−^ in Ag_3_PO_4_ and PAN in SAP/PAN was confirmed by performing XPS analysis. As shown in survey spectrum (Figure 5a), P, S, Ag, and O elements coming from SO_4_^2−^ -Ag_3_PO_4_ and C and N elements corresponding to PAN were clearly observed. Moreover, specific nature of S in SAP/PAN and Ag, P, and O in both samples was obtained from high-resolution XPS spectra. As depicted in Figure 5b, a peak located at around 168.38 eV in high-resolution spectra of S 2p in SAP/PAN was attributed to S^6+^ [37]. This result also indicated the incorporation of SO_4_^2−^ into Ag_3_PO_4_ crystal lattice during synthesis process. In case of Ag 3d and P 2p peaks of SAP/PAN, slight shifting of these peaks to higher values of binding energies was observed compared to that of AP/PAN (Figure 5c,d). This shifting might happen due to doping of SO_4_^2−^, which could decrease electron density around Ag and P due to higher electronegativity of S [38]. Similar behavior was observed for O 1s peak of SAP/PAN compared to AP/PAN (Figure 5e). Hence, all these XPS results further confirmed the existence of S in the form of SO_4_^2−^ in Ag_3_PO_4_ crystal lattice due to its strong electronic interactions with Ag, P, and O [19].

**Figure 6:**
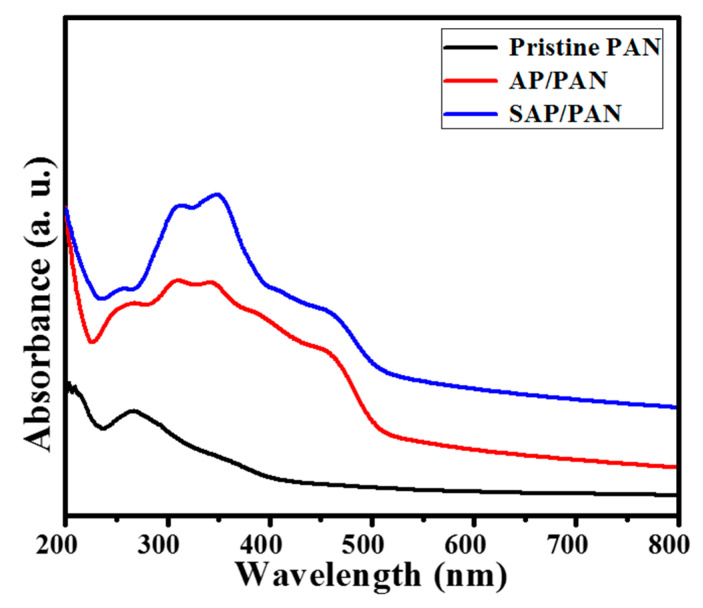
UV-vis diffusive reflectance spectra of prepared photocatalysts.

UV-vis diffusive reflectance spectra (DRS) of pristine PAN, AP/PAN, and SAP/PAN were measured to determine their light absorption behavior and the results are plotted in Figure 6. As seen, two absorption bands presented in the range of 200–350 nm were assigned to the pristine PAN, which is in agreement with the result of a previously reported study [39]. After loading sulfate undoped/doped nanoparticles on PAN nanofibers, visible light absorption behavior could be observed. Both the samples (AP/PAN and SAP/PAN) displayed continuous absorption in visible range (520–700 nm), however the absorption intensity of SAP/PAN was found to be slightly increased. Therefore, these results signified the visible light harvesting capability of AP/PAN and SAP/PAN.

**Figure 7:**
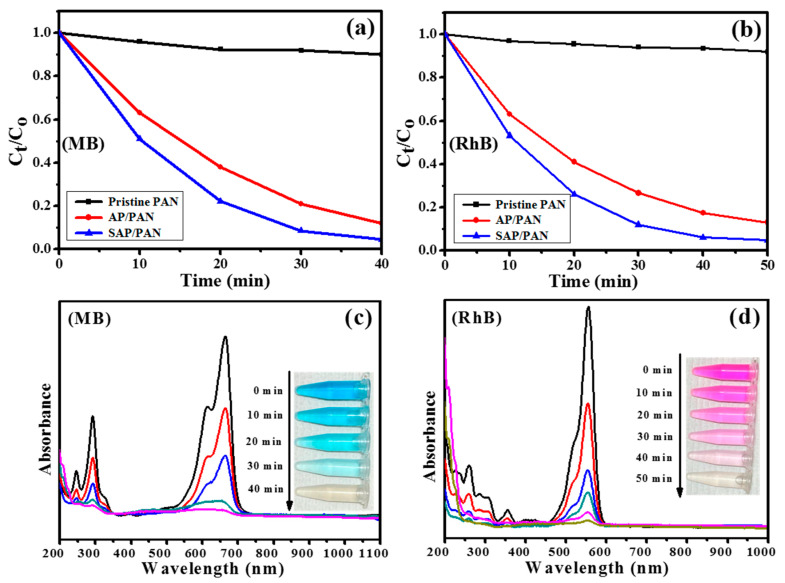
Photocatalytic performances of pristine PAN, AP/PAN, and SAP/PAN towards photodegradation of MB (**a**) and RhB (**b**) solutions. Absorbance variation of MB (**c**) and RhB (**d**) solutions utilizing SAP/PAN. Insets; digital photographs showing the color change of corresponding dye solution with irradiation time.

Photodegradation performances of different samples under visible light irradiation were investigated using MB and RhB dye solutions and results are presented in Figure 7a,b. The photodegradation is represented as the variation of (C_t_/C_o_) with irradiation time, where C_o_ is the initial concentration and C_t_ is remaining concentration of dyes solution at time t. As presented in figure, pristine PAN could show negligible capability of photodegradation of MB and RhB dye solutions. In contrary, more than 95% of MB was degraded by SAP/PAN within 40 min, while only 88% of MB was degraded within this time period utilizing AP/PAN. Likewise, SAP/PAN could exhibit superior performance over AP/PAN towards the photodegradation of RhB. In this case, SAP/PAN could degrade 95% of RhB within 50 min, but within this time period, AP/PAN could degrade about 87% of RhB. On the basis of these results, SAP/PAN was found to be a more advantageous visible-light-driven photocatalyst over AP/PAN. Figure 7c,d show the time-dependent absorbance variations of MB and RhB dye solutions utilizing SAP/PAN under visible light irradiation. Corresponding absorbance peaks of MB at 665 nm and RhB at 554 nm are gradually diminished with the increase in irradiation time. Importantly, the maximum wavelength of MB and RhB were not found to be shifted, which indicated that benzene/heterocyclic rings were decomposed rather than decolorized due to adsorption of dye molecules on the surface of photocatalyst [40,41]. Insets (Figure 7c,d) show gradual decline in color of corresponding dye solution utilizing SAP/PAN.

**Figure 8:**
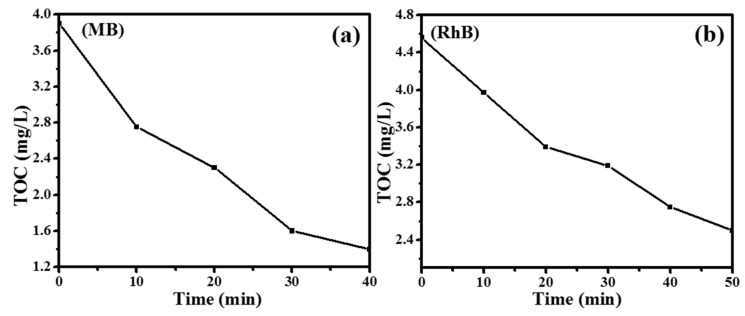
Changes in total organic carbon (TOC) during photodegradation of (**a**) MB and (**b**) RhB with SAP/PAN.

The changes of TOC during photodegradation of MB and RhB utilizing SAP/PAN under visible light irradiation are displayed in Figure 8. As displayed in the figure, TOC of MB with SAP/PAN after 10, 20, 30, and 40 min of irradiation were 2.75, 2.3, 1.6, and 1.4 mg/L, respectively. Similarly, TOC of RhB with SAP/PAN after 10, 20, 30, 40, and 50 min of irradiation were 3.97, 3.39, 3.19, 2.75, and 2.5 mg/L, respectively. These results showed that TOCs were lower than that of original dye solutions (MB = 3.9 mg/L and RhB = 4.56 mg/L). Furthermore, the rate of TOC change for both MB and RhB dyes was lower compared to their photodegradation rate, which is assigned to the partial decomposition of dye molecules into intermediate products resulting in the disappearance of color and partial mineralization [42,43].

**Figure 9:**
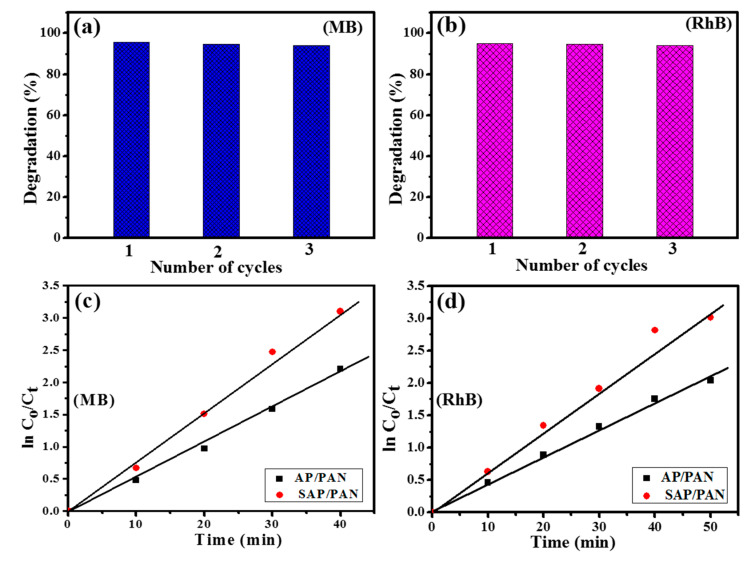
Cycling experiments of photodegradation of MB (**a**) and RhB (**b**) solutions utilizing SAP/PAN. Photodegradation Kinetics of MB (**c**) and RhB (**d**) solutions over different photocatalysts under visible light irradiation.

Photodegradation stability of SAP/PAN was examined by performing cycling experiments for MB and RhB degradation under visible light irradiation (Figure 9a,b). For cycling experiments, the used sample was separated, washed, and dried at room temperature then reapplied for photodegradation under similar conditions. Experimental results showed good stability of SAP/PAN up to third cycle, however slight decrease was observed in the performance during cycling experiments, which could happen due to loss of photocatalyst during separation process. Furthermore, Langmuir–Hinshelwood model was applied to evaluate the photodegradation kinetics of MB and RhB solutions utilizing AP/PAN and SAP/PAN.

(1) $$r=-\frac{dc}{dt}=\frac{k_{r}KC}{1+KC}.$$

Since the initial concentration of MB and RhB was very low (C_o_ = 10 mg/L), equation (1) can be considered a pseudo first-order kinetics equation [44] as

(2) $$lnC_{o}/C_{t}=k_{app}t,$$

where C_o_ and C_t_ represent the initial concentration and concentration at time (t), respectively. k_app_ is the apparent rate constant (min^−1^), which can be obtained by plotting ln (C_o_/C_t_) vs. reaction time. Hence, degradation kinetics of MB and RhB solutions were calculated by applying equation (2) and the results are shown in Figure 9c,d. The linear relationship between ln (C_o_/C_t_) vs. reaction time suggested the pseudo first-order kinetics of photodegradation. From the results, apparent rate constants of MB degradation utilizing AP/PAN and SAP/PAN were determined to be 0.057 min^−1^ and 0.075 min^−1^, respectively. Similarly, the apparent rate constants 0.043 min^−1^ and 0.064 min^−1^ were determined for RhB degradation utilizing AP/PAN and SAP/PAN, respectively. All these results indicated that sulfate doping could provide significant capability to Ag_3_PO_4_ to enhance its photodegradation performance.

On the basis of above results, an overall mechanism is proposed for photodegradation of organic dyes. Ag_3_PO_4_, being a semiconductor material, generates electron–hole pairs under visible light irradiation. The photo-excited electrons travel to conduction band (CB) from valence band (VB) and react with dissolved oxygen molecules to produce ROS, i.e., oxygen peroxide radicals (O_2_^• −^), which are strong oxidizing agents and degrade dye molecules effectively. On the other hand, holes at VB directly react with dye molecules [45]. In this way, these ROS and holes can photocatalytically degrade organic dyes as

Organic dyes + O_2_^• −^ → CO_2_ + H_2_O + mineralization products. (3)

**Figure 10:**
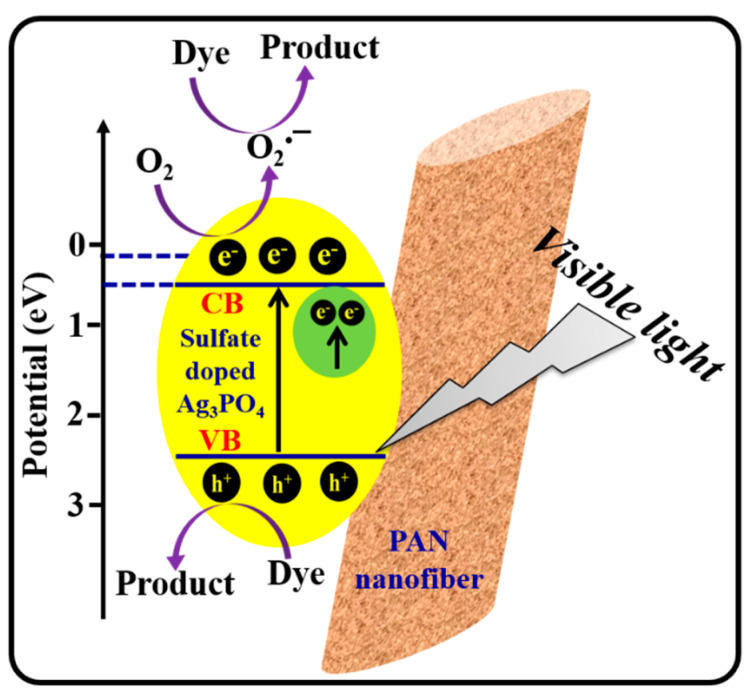
Schematic illustration for the degradation of dye by sulfate-doped Ag_3_PO_4_/PAN under visible light irradiation.

It is well known that semiconductor photocatalysts having improved separation capability and low recombination rate of photoinduced electron–hole pairs can exhibit enhanced performances. Hence, in this work the enhanced photocatalytic performances of SAP/PAN compared to that of AP/PAN can be explained with the role of SO_4_^2−^ as dopant, which could play an important role to trap and transfer photoinduced electrons to CB, thereby providing improved separation capability of electron–hole pairs to SO_4_^2−^-Ag_3_PO_4_ (Figure 10). Moreover, SO_4_^2−^-Ag_3_PO_4_ could receive additional electrons due to higher electronegativity of S than that of P. As a result, the Fermi level of SO_4_^2−^-Ag_3_PO_4_ gets shifted towards CB and possesses n-type conductivity. In this way, doping of SO_4_^2−^ into the Ag_3_PO_4_ crystal lattice can improve its separation capability and lower recombination rate of photoinduced electron–hole pairs, which ultimately increases the production of ROS and enhances the photocatalytic performance of SO_4_^2−^-Ag_3_PO_4_ [22,46,47].

## 4. Conclusions

In summary, SO_4_^2−^-Ag_3_PO_4_/PAN-electrospun nanofibers were fabricated successfully by combining electrospinning and ion-exchange reaction. Different characterization techniques were used to study the morphology, structure, chemical composition, and optical properties of the samples. Photocatalytic activity of the fabricated samples was investigated by photodegradation of MB and RhB dye solutions under visible light irradiation. In both investigations, SO_4_^2−^-Ag_3_PO_4_/PAN-electrospun nanofibers could show enhanced performance compared to Ag_3_PO_4_/PAN-electrospun nanofibers. We believe that the enhanced performances of SO_4_^2−^-Ag_3_PO_4_/PAN-electrospun nanofibers were attributed to the sufficient electron–hole separation capability of SO_4_^2−^-Ag_3_PO_4_ nanoparticles to produce ROS due to doping effect of SO_4_^2−^ ions into the Ag_3_PO_4_ crystal lattice. Therefore, thus fabricated SO_4_^2−^-Ag_3_PO_4_/PAN-electrospun nanofibers can find potential application as visible-light-driven photocatalyst with good flexibility and reusability for wastewater treatment.
